# Proteomics and transcriptomics profiling reveals distinct aspects of kidney stone related genes in calculi rats

**DOI:** 10.1186/s12864-023-09222-7

**Published:** 2023-03-17

**Authors:** Wang Zhu, Deng Qiong, Gu Yanli, Li Min, Zhang Ying, Hu Qiyi, Zhang Shenping, Wang Xisheng, Liang Hui

**Affiliations:** 1 Department of Urology, People’s Hospital of Longhua Shenzhen, Southern Medical University, 38 Jinglong Jianshe Road, Shenzhen, Guangdong 518109 People’s Republic of China; 2 Central Laboratory, People’s Hospital of Longhua Shenzhen, Southern Medical University, Shenzhen, Guangdong 518109 People’s Republic of China; 3 Department of Pathology, People’s Hospital of Longhua Shenzhen, Southern Medical University, Shenzhen, Guangdong 518109 People’s Republic of China

**Keywords:** Calcium oxalate, Kidney stone, Proteomics, Transcriptomics, Urolithiasis

## Abstract

**Backgrounds:**

Kidney stone also known as urolithiasis or nephrolithiasis, is one of the oldest diseases known to medicine, however, the gene expression changes and related kidney injury remains unclear.

**Methods:**

A calculi rat model was developed via ethylene glycol– and ammonium chloride–induction. Integrated proteomic and transcriptomic analysis was performed to characterize the distinct gene expression profiles in the kidney of calculi rat. Differential expressed genes (DEGs) were sub-clustered into distinct groups according to the consistency of transcriptome and proteome. Gene Ontology and KEGG pathway enrichment was performed to analyze the functions of each sub-group of DEGs. Immunohistochemistry was performed to validated the expression of identified proteins.

**Results:**

Five thousand eight hundred ninety-seven genes were quantified at both transcriptome and proteome levels, and six distinct gene clusters were identified, of which 14 genes were consistently dysregulated. Functional enrichment analysis showed that the calculi rat kidney was increased expression of injured & apoptotic markers and immune-molecules, and decreased expression of solute carriers & transporters and many metabolic related factors.

**Conclusions:**

The present proteotranscriptomic study provided a data resource and new insights for better understanding of the pathogenesis of nephrolithiasis, will hopefully facilitate the future development of new strategies for the recurrence prevention and treatment in patients with kidney stone disease.

**Supplementary Information:**

The online version contains supplementary material available at 10.1186/s12864-023-09222-7.

## Introduction

Kidney stone, also known as urolithiasis or nephrolithiasis, is one of the oldest diseases known to medicine, causing systemic symptoms, including endocrine disorders, metabolic syndrome, chronic kidney insufficiency [1], autoimmune diseases, osteoporosis [2], inflammatory diseases, hypertension and most recently ischemic strokes [3–6]. The prevalence of kidney stone disease is steadily increased worldwide in the recent decades. Previous literature using National Health and Nutrition Examination Survey (NHANES) data reported that the prevalence of kidney stone has continued to rise in the United States from 3.2% in 1980 [7] to 5.2% in 1994 [7, 8], 8.8% in 2010 and 10.1% in 2016 [9]. There is a high probability of recurrence of urinary stones, estimated to be up to 52% within 10 years [10, 11].

Benefit from technologies development, a large number of genes and proteins have been identified, which are reported to be involved in the process of kidney stone formation. Several macromolecules, such as Spp1, the vitamin K-dependent protein matrix Gla protein (MGP), Bakunin [8], and Tamm-Horsfall proteins (THP) [9], have been identified in both the urine and kidney stone matrix affect the risk of kidney stone disease [10–12]. RNA sequencing studies demonstrated that a large number of coding or non-coding RNAs were dysregulated expression in the kidneys of calculi rats, which involved in complement and coagulation cascades, cytokine-cytokine receptor interactions, ECM-receptor interactions and histidine metabolism [12, 13]. Recently, we have identified a total of 1 141 proteins by TMT-labeled quantitative proteomics analysis, of which 699 were up-regulated and 442 were down-regulated in the calcium oxalate monohydrate (COM)-crystal treated HK-2 cells [14]. These proteins play role in modulating COM crystal initiation, provide us with amount of the possible signaling pathways, potential targets and interaction networks for understanding of the pathogenesis of kidney stones.

However, the aforementioned studies mostly focused on the transcripts or protein level dysregulations of the genes related to stone formation. Mounts of genes exhibited inconsistent expression patterns in mRNA level and protein level have been largely neglected. Accumulated studies indicated that CaOx induced endoplasmic reticulum (ER) stress mediated posttranslational protein modification also play a critical role in the gene expression related to kidney stone disease [15–17]. Achievements have been made to uncover the post-translational-related molecular mechanisms of nephrolithiasis, but more investigations are needed based on advances in technologies and bioinformatics.

## Material and methods

### Experiment design and sample collection

All animal experiments were performed with adult male Sprague–Dawley (SD) rats (250–300 g), in accordance with the guidelines for the care and use of laboratory animals, and approved by the ethics committee of People’s Hospital of Longhua Shenzhen (LHRY-1907015). The rats were maintained and habituated in a standard 12-h light–dark cycle with ad libitum access to food and water in a temperature and humidity-controlled room, maintaining 22 °C ± 0.5 °C and a relative humidity of 40–60%. SD rats were randomly divided into a control group and kidney stone group. The control group only received normal rat chow and sterile water for 14 days. The kidney stone group received drinking water with 1% (*v/v*) ethylene glycol (EG, Sigma-Aldrich, Buchs, Switzerland) and 1% (*w/v*) ammonium chloride 1 ml per day by gavage for 3 weeks. Bilateral kidneys of the rats were removed under 4% isoflurane (CAS:26,675–46-7, RWD, Shenzhen, China) inhalation anesthesia for 3 min. The rats were then sacrificed via cervical dislocation after CO_2_ sedation. One kidney per rat was fixed in 4% paraformaldehyde, dehydrated in ethanol solution, embedded into paraffin, sliced into 5-μm serial sections, stained with Hematoxylin–Eosin (HE) and von-Kossa’s staining, and observed to detect CaOx crystals using a polarizing microscope. The other kidney was applied for RNA and protein extraction.

### RNA preparation, cDNA synthesis and RNA sequencing

Total RNA was extracted using TRIzol method. RNA purity was checked using the NanoPhotometer® spectrophotometer (IMPLEN, CA, USA). RNA concentration was measured using Qubit® RNA Assay Kit in Qubit® 2.0 Flurometer (Life Technologies, CA, USA). RNA integrity was assessed using the RNA Nano 6000 Assay Kit of the Bioanalyzer 2100 system (Agilent Technologies, CA, USA). Sequencing libraries were generated using NEBNext® Ultra™ RNA Library Prep Kit for Illumina® (NEB, USA) following manufacturer’s recommendations and index codes were added to attribute sequences to each sample. The clustering of the index-coded samples was performed on a cBot Cluster Generation System using TruSeq PE Cluster Kit v3-cBot-HS (Illumia) according to the manufacturer’s instructions. After cluster generation, the library preparations were sequenced on an Illumina Hiseq2500/X platform and 125/150 bp paired-end reads were generated. The FPKM of all genes has been provided in Supplementary Table 1.

### Protein preparation and proteomics

The protein extraction and quality analyzation were performed according to previously reports [14]. In short, the sample was grinded by liquid nitrogen into cell powder and then transferred to a 5-mL centrifuge tube. After that, four volumes of lysis buffer (8 M urea, 1% Protease Inhibitor Cocktail) was added to the cell powder, followed by sonication three times on ice using a high intensity ultrasonic processor (Scientz). The remaining debris was removed by centrifugation at 12,000 g at 4 °C for 10 min. Finally, the supernatant was collected and the protein concentration was determined with bicinchoninic acid (BCA) kit according to the manufacturer’s instructions.

Take equal amount protein of each sample, and adjust to equal volume with lysis buffer for digestion according to previous studies [18, 19]. The protein solution was reduced with 5 mM dithiothreitol for 30 min at 56 °C and alkylated with 11 mM iodoacetamide for 15 min at room temperature in darkness. The protein sample was then diluted by adding 100 mM triethyl-ammonium bicarbonate buffer (TEAB) to urea concentration less than 2 M. Finally, trypsin was added at 1:50 trypsin-to-protein mass ratio for the first digestion overnight and 1:100 trypsin-to-protein mass ratio for a second 4 h-digestion.

The liquid chromatograph-mass spectrometer (LC–MS/MS) analysis was performed by PTM Biolabs Inc (Hangzhou, China) according to previous studies [19]. In short, the tryptic peptides were dissolved in solvent A (0.1% formic acid, 2% acetonitrile/in water), directly loaded onto a home-made reversed-phase analytical column (25-cm length, 75/100 μm i.d.). Peptides were separated with a gradient from 6 to 24% solvent B (0.1% formic acid in acetonitrile) over 70 min, 24% to 35% in 14 min and climbing to 80% in 3 min then holding at 80% for the last 3 min, all at a constant flow rate of 450 nL/min on a nanoElute UHPLC system (Bruker Daltonics) [19].

The peptides were subjected to capillary source followed by the timsTOF Pro (Bruker Daltonics) mass spectrometry as reported previously [20]. The electrospray voltage applied was 1.60 kV; Precursors and fragments were analyzed at the TOF detector, with a MS/MS scan range from 100 to 1700 m/z; The timsTOF Pro was operated in parallel accumulation serial fragmentation (PASEF) mode; Precursors with charge states 0 to 5 were selected for fragmentation, and 10 PASEF-MS/MS scans were acquired per cycle; The dynamic exclusion was set to 30 s [20].

The secondary mass spectrometry data were retrieved using MaxQuant (v1.6.15.0). Tandem mass spectra were searched against the database of Rattus_norvegicus_10116_Ensembl_Rnor_6.0_20210527.fasta (including 17,063 sequences) concatenated with a reverse decoy database. The cleavage enzyme was specified as Trypsin/P. The maximum number of missed cleavages per peptide was set as 2. The mass tolerance for precursor ions in the first search was set to 20 parts per million (ppm) and 5 ppm in the main search, and the mass tolerance for fragment ions was set at 0.05 Da. The cysteine alkylation Carbamidomethyl (C) was specified as a fixed modification, and the oxidation on methionine (Met) residues, acetylation on proteins N-termini were specified as variable modifications.

The false discovery rate (FDR) was adjusted to < 1% for both proteins and peptides. The identified protein should contain at least one unique peptide. The MS identification information has been described in Supplementary Table 2.

### Bioinformatics analysis

Differential expression analysis of the two groups was performed using the DESeq2 R package. The resulting *P*-values were adjusted using the Benjamini and Hochberg’s approach for controlling the false discovery rate. Genes with an adjusted *P* < 0.01 found by DESeq2 were assigned as differentially expressed. Gene Ontology (GO) and Kyoto Encyclopedia of Genes and Genomes (KEGG, www.kegg.jp/kegg/kegg1.html) pathway enrichment analysis of differentially expressed genes (DEGs) was implemented by the GOseq R package, in which gene length bias was corrected. GO terms with corrected *P* < 0.05 were considered significantly enriched by DEGs. We used KOBAS software to test the statistical enrichment of DEGs in KEGG pathways with the permission of Kanehisa Laboratories [21, 22].

### Histopathological analysis

Peroxidase immunohistochemistry of target proteins were performed using specific antibodies, anti-HAVCR1(Abcam, ab233720), Anti-C5(Abcam, ab275931), anti-AKR1B8(Thermofisher, PA5114302), anti-Spp1(Proteintech Group, 25,715–1-AP), anti-C3(Proteintech Group, 21,337–1-AP), anti-VILL(Novus, NBP2-86,054), anti-HAAO(Proteintech Group, 12,791–1-AP), anti-TEMT(Abcam, ab181854), anti-CSAD(Abcam, ab91016), anti-ALK(Abcam, ab203106), anti-MIOX(Abcam, ab154639), anti-GPX2(Abcam, ab137431), anti-ASRGL1(Proteintech, 11,400–1-AP) and anti-TUBB6(Proteintech, 66,362–1-Ig) following procedures as described previously [23]. The histogram profile and score of a cytoplasmic and nuclear stained immunohistochemistry image was determined by ImageJ program (1.8.0 version) with the IHC profiler plugin as described previously [24–26]. The quantified immunoscore was entered into an Excel spreadsheet and analyzed by GraphPad Prism 8.

## Results

### Model development and sample qualification

**Fig. 1:**
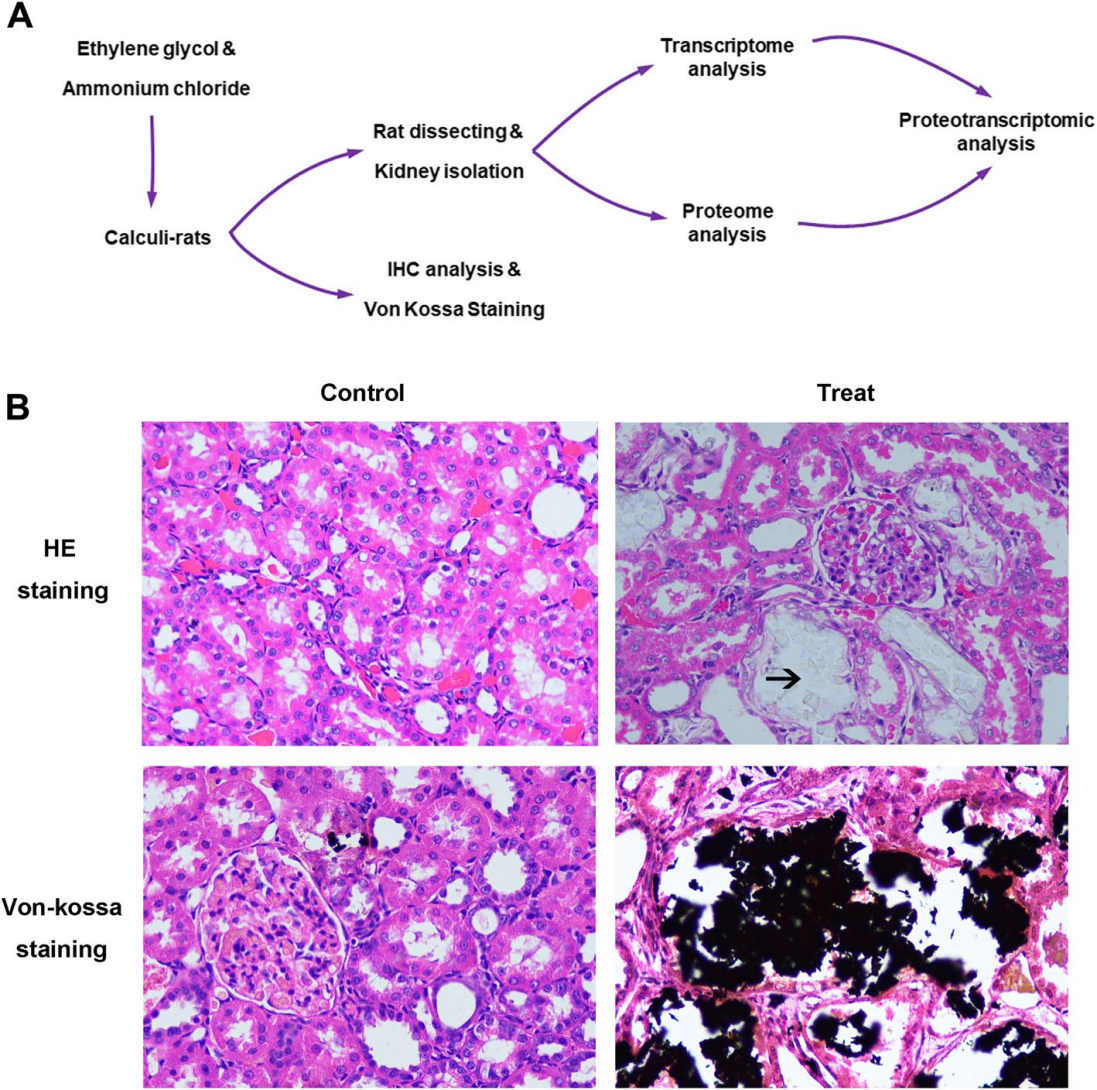
Study design and validation of the calculi rat model. **A** Study design and workflow of rat kidney sample processing for proteomics and transcriptomics analysis. **B** Histochemical validation of the calcium crystals in the calculi rat model via HE staining and von Kossa’s staining. The arrows indicate the calculi oxalate crystals. Original magnification, × 40

We developed the urolithiasis model by ethylene glycol and ammonium chloride-induced SD rats, followed by integrative RNA-seq transcriptomic and label-free proteomic experiments (Fig. 1A). Before the analysis, HE and von Kossa's staining was performed to detect CaOx deposits and tubulointerstitial damages of the rat kidney. In the experimental group (*n* = 3), a mount of CaOx deposits were found inside the proximal tubules, loops of Henle, distal tubules and collecting ducts. Considerable tubulointerstitial damages such as tubular atrophy, dilation, hyaline cast, tubular cell necrosis and interstitial inflammation were observed in renal tissue of calculi rats (Fig. 1B).

**Fig. 2:**
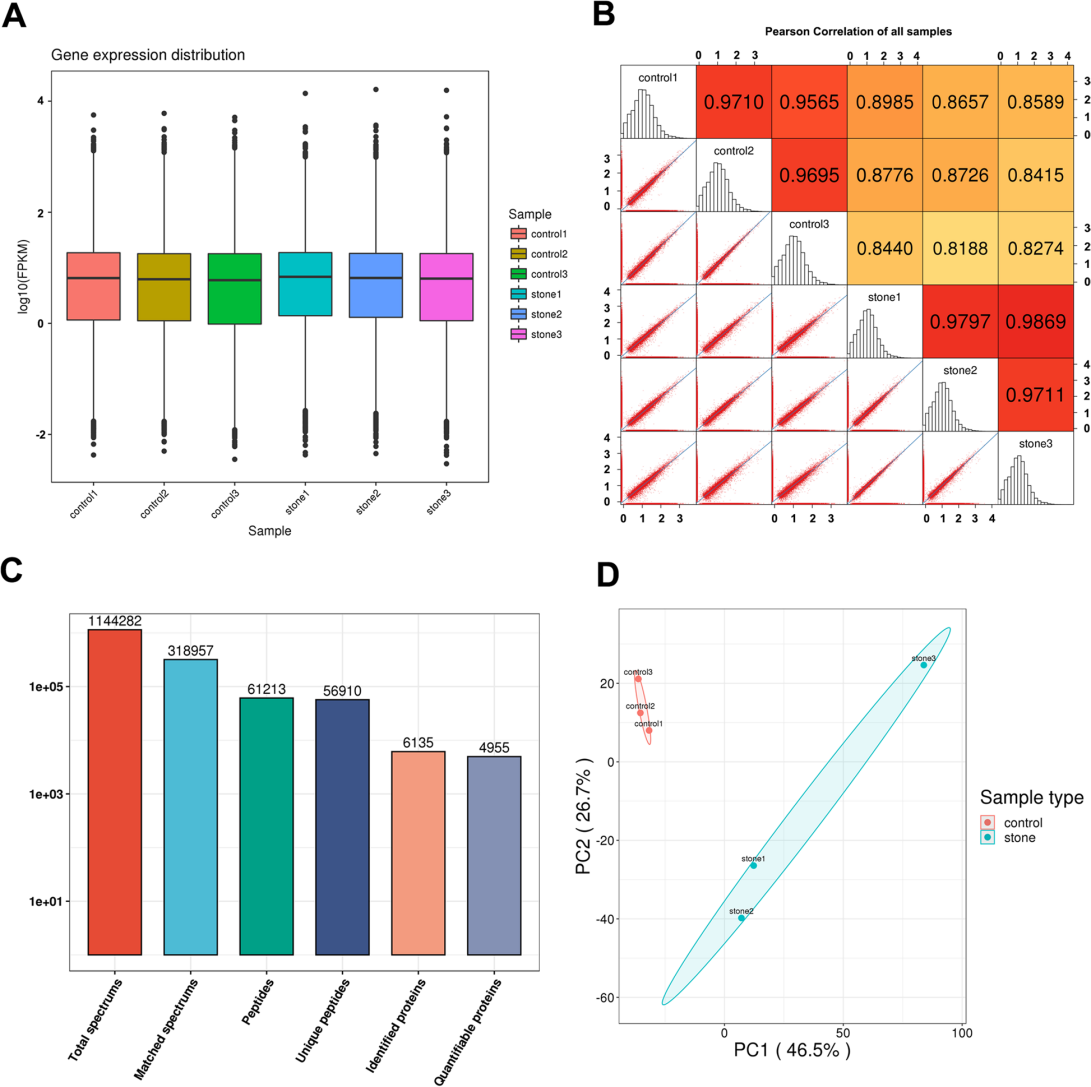
Sample qualification. **A** FPKM box diagram. The abscissa in the figure represents different samples. The ordinate represents the logarithm of the sample expression FPKM. The figure measures the expression level of each sample from the perspective of the overall dispersion of the expression amount. **B** The Pearson correlation of all samples. **C** Overview of protein identification. **D** Principal component analysis (PCA) to evaluate the repeatability of protein quantification

We firstly showed the dispersion of the gene expression level distribution of samples in both treated and control groups. The overall gene expression level of different samples showed no significant differences (Fig. 2A). Pearson's correlation coefficient R was used to evaluate the correlation between different groups. Results showed stronger correlation between biological replicates than different groups, suggested significant difference of gene expression profiles between control and treat groups (Fig. 2B). For protein identification, we got 1 144 282 total spectrums, and then identified 6 135 proteins, of which 4 955 proteins quantifiable (Fig. 2C). Principal component analysis (PCA) indicated that better quantitative repeatability of biological replicates than different groups (Fig. 2D). These results demonstrated that the CaOx calculi rat model was developed successfully, and there was significant difference of gene expression profiles between control and treat groups.

### Overview of transcriptomic analysis

**Fig. 3:**
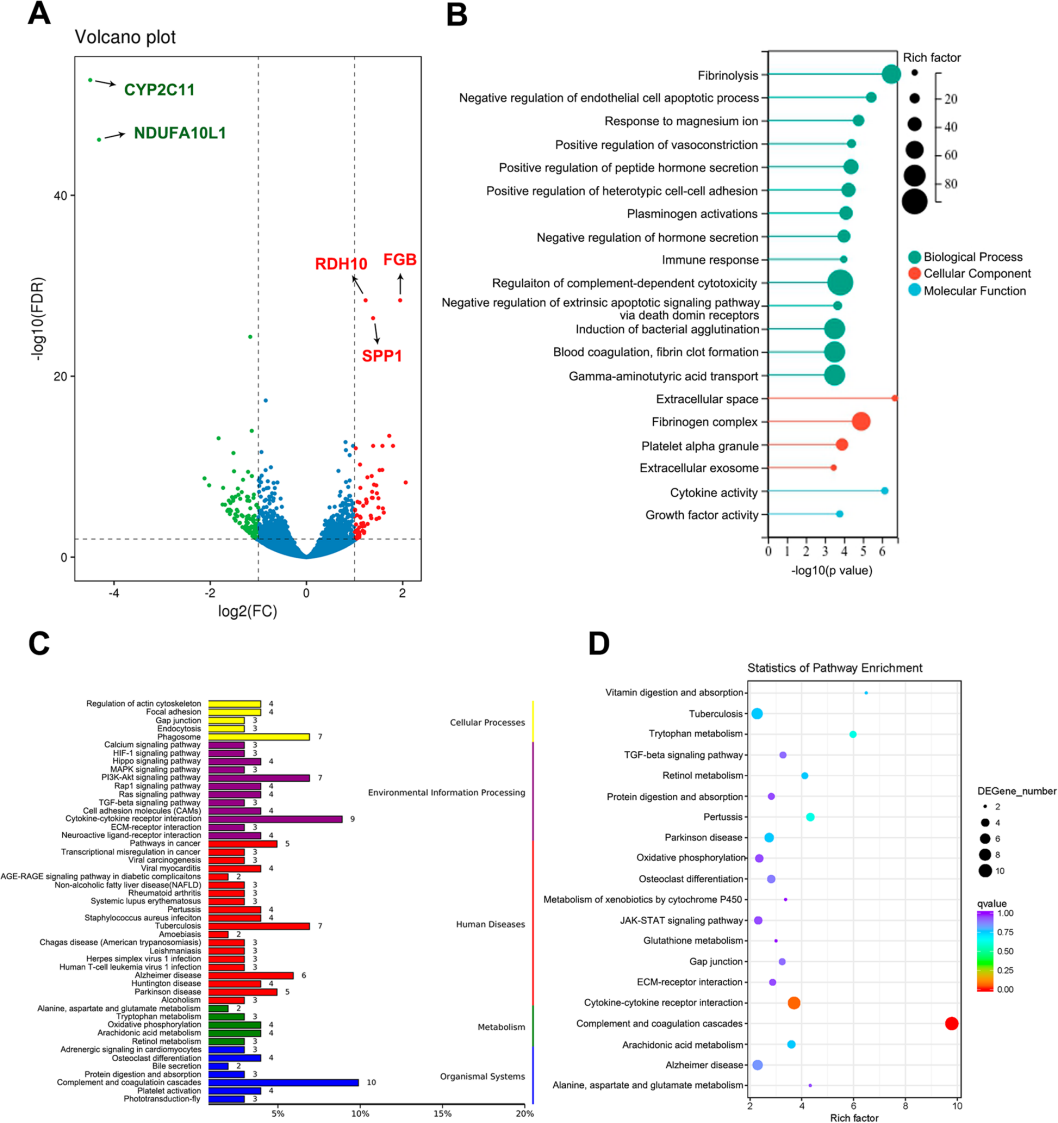
Overview of transcriptomic analysis. **A** Volcano plot of differential expressed genes in the kidney of calculi rat. **B** GO terms enrichment of the DEGs. **C** KEGG enrichment analysis of differential gene. **D** Statistics of pathway enrichment

We found that 191 genes were dysregulated in the kidney of calculi rats, of which 72 genes were up-regulated and 119 genes were down-regulated (Fig. 3A, Supplementary Table 3). Notably, the Gprin3 and Fgb were the most up-regulated genes in the kidney of calculi rats. Gprin3 is belonged to the G-protein-regulated inducer of neurite outgrowth (GPRIN) family, acting as a partner of β-arrestin-2, regulating dopamine receptor desensitization and playing roles in striatal physiology [27, 28]. Nevertheless, the biological roles of GPRIN3 in kidney stone formation is still unknown. Emerging kidney injury biomarkers such as Lcn2 and Havcr1 are also significantly increased expression in the kidney tissue of calculi rats. Spp1 also showed significantly increased expression in the kidney of calculi rats, which is an immunoregulatory molecule for immune cells, in particular, for neutrophils and macrophages and enhances T helper 1 inflammation [29]. A recent study demonstrated that Spp1 serum levels were correlated with kidney injury, and facilitated with AKI–induced acute lung injury (ALI) [30]. Therefore, our data indicates substantial kidney injury occurred at molecular and genetic level in the calculi rats.

GO annotation and enrichment analysis were conducted to identify essential terms associated with the formation of calculi, as well as calculi-mediated kidney injury. The results showed that in the top 20 most enriched GO terms, 14 terms are related to the biological processes. Four cellular component terms, including extracellular space, extracellular exome, fibrinogen complex and platelet alpha granule were enriched. Two molecular function terms, cytokine activity and growth factor activity were enriched (Fig. 3B). In addition, KEGG enrichment analysis was conducted to evaluate the important signaling pathways of the differential expressed genes (DEGs). Results showed that the complement and coagulation cascades (KO:04,610) and cytokine-cytokine receptors interaction (KO:04,060) were most enriched (Fig. 3C, D). These findings suggested that the immunoregulatory features might have been significantly changed in the kidney of calculi rats.

### Proteomic profiling of the kidney in calculi rats

**Fig. 4:**
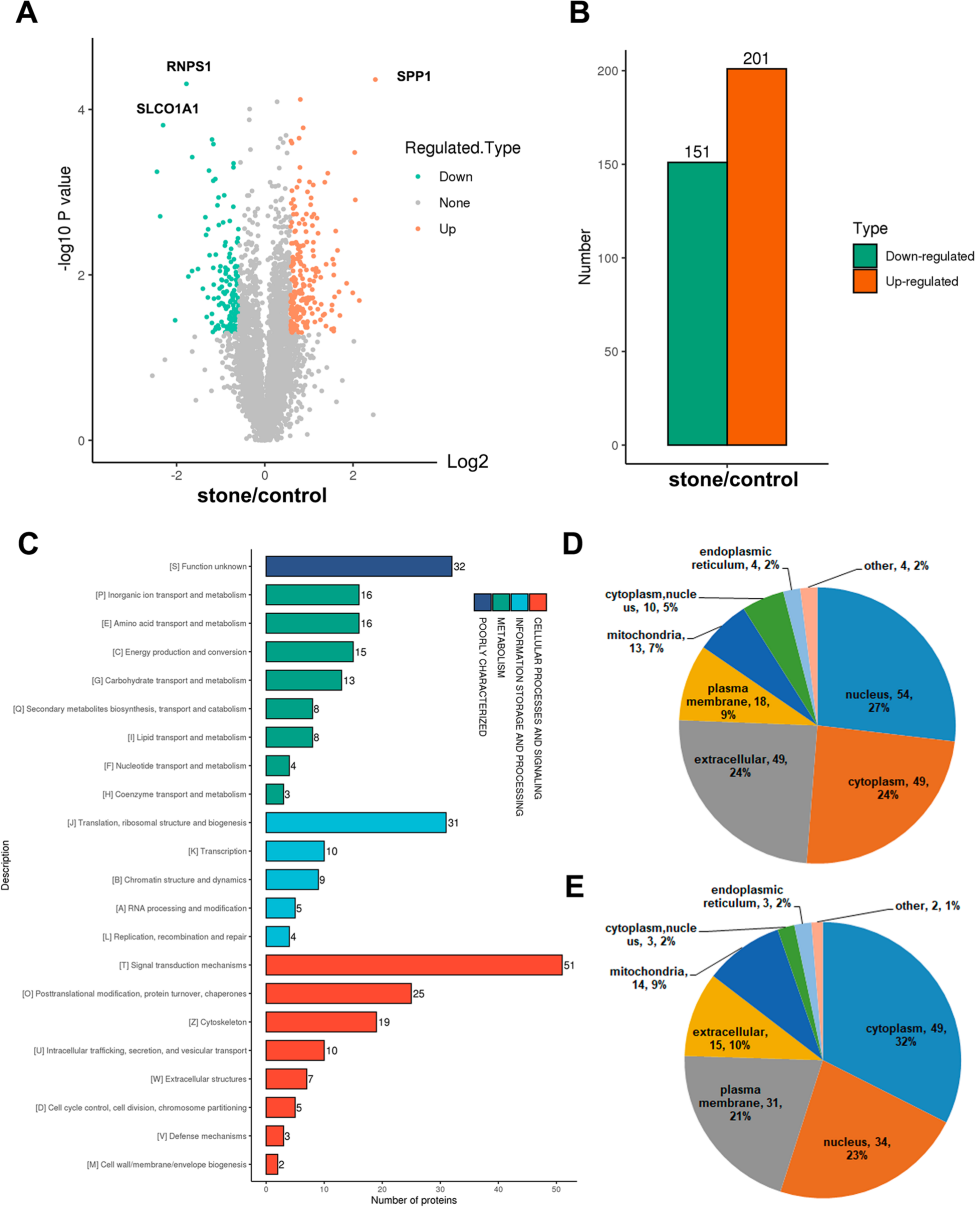
Proteomic profiling of the kidney in calculi rats. **A** Volcano plot of differential expressed proteins. **B** Statistical chart of differential expressed proteins. **C** GO function classification. **D** Subcellular annotation of up-regulated proteins. **E** Subcellular annotation of down-regulated proteins

Among the 4 955 quantifiable proteins, we identified totally 352 differential expressed proteins (DEPs) in the kidney of calculi rat, of which 201 proteins were up-regulated and 151 proteins were down-regulated (Fig. 4A, B). The hierarchical clustering heatmap of the DEGs and DEPs was showed in Supplementary Fig. 1. Notably, the kidney injury-related molecules such as Havcr1 and Spp1, and apoptosis-related markers such as Niban1, Casp1, Casp3 and Casp8 were significantly up-regulated in the kidney of calculi rat compare to its normal control (Supplementary Table 4). Nevertheless, multiple important solute carriers (Slc12a6, Slc33a1, Slc15a2, Slc43a2, Slc8a1, Slc22a2, Slc22a1, Slc22a6, Slc22a8, Slc22a22, Slc6a8, Slc2a1, Slc37a4 and Slc34a1) and components of vacuolar ATPase (Atp6v1b1, Atp6v0a4, Atp6v1g3, Atp6v1c2) were decreased expression in the kidney of calculi rats (Supplementary Table 4), which indicated that decline of kidney function in calculi rats. We also found that the CD44 (Spp1 receptor) and a number of complements (C1qb, C1qc, C2, C3, C5, C6, C7, C8a, C8b and C9) were significantly increased in the kidney of calculi rats. It’s well known that Spp1:CD44 signaling is critical for GPCR-mediated chemotaxis of neutrophils and macrophage, which is required for the development of cell-mediated inflammatory responses [31, 32]. Our data suggested that complements-related immunoregulatory might play an important role in calculi-mediated kidney injury.

**Fig. 5:**
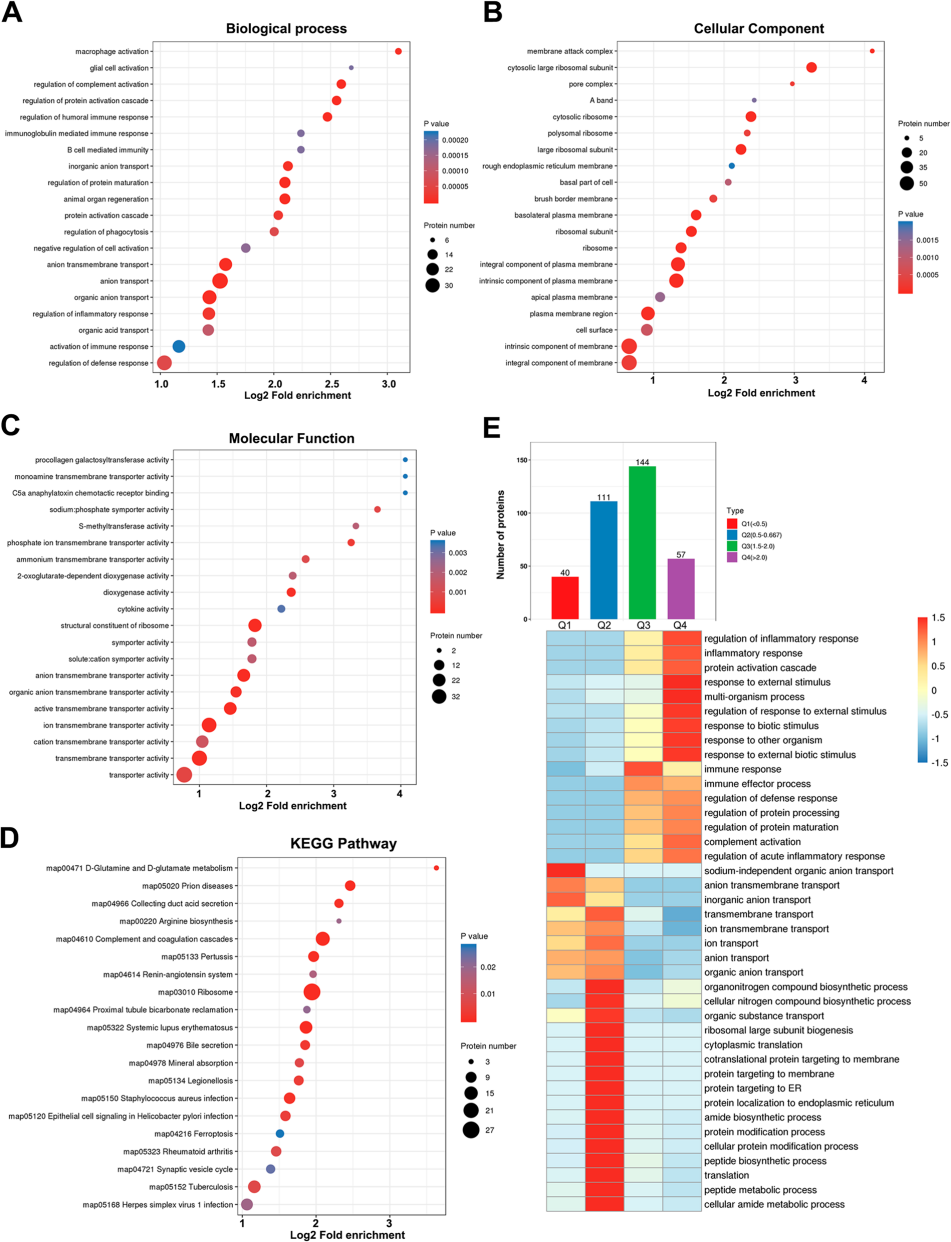
Functional enrichment analysis of differentially expressed proteins. **A** Biological process. **B** Cellular component. **C** Molecular function. **D** KEGG pathway. **E** Clustering analysis. Up: the differential expressed proteins were divided into four groups (Q1 to Q4) according to their expression level. Down: heatmap of the biological process terms of each subgroup. The color bar indicates the enrichment degree

GO category analysis was conducted to evaluate the critical terms of the DEPs involved in kidney stone formation. Results showed that most of the DEPs were involved in signal transduction mechanisms (51 proteins), and translation, ribosomal structure and biogenesis (31 proteins) (Fig. 4C). Interestingly, the proteins (44 proteins) related to signal transduction were significantly up-regulated, while proteins associated with translation, ribosomal structure and biogenesis (27 proteins) were down-regulated (Supplementary Fig. 2). Subcellular location analysis showed that most of the up-regulated proteins were located in the nucleus (27%), and then cytoplasm (24%) and extracellular (24%) (Fig. 4D). However, most of the down-regulated proteins were located in the cytoplasm (32%) and nucleus (23%) (Fig. 4E). Functional enrichment analysis indicated that macrophage activation was the most enriched terms of biological process (Fig. 5A). Membrane attack complex was the most enriched cellular component (Fig. 5B), and Symporter activity, transporter activity and cytokine activity were the most enriched molecular function terms (Fig. 5C). KEGG pathway enrichment analysis showed that most DEPs were involved in ribosome, and complement and coagulation cascades (Fig. 5D).

We then divided the DEPs into four parts according to its differential expression level, marked as Q1 to Q4, as showed in the Fig. 5E. For each group, we performed GO enrichment and cluster analysis to find the correlation between protein functions and differential expression levels. Q4 cluster includes 57 most up-regulated proteins, which was highly correlated with cell response to external stimulus and inflammatory response. The Q1 was a cluster includes 40 most down-regulated proteins mainly related to sodium-independent organic anion transport and inorganic anion transport (Fig. 5E).

### Identification of distinct gene expression profiles by integrated proteomic and transcriptomic analysis

**Fig. 6:**
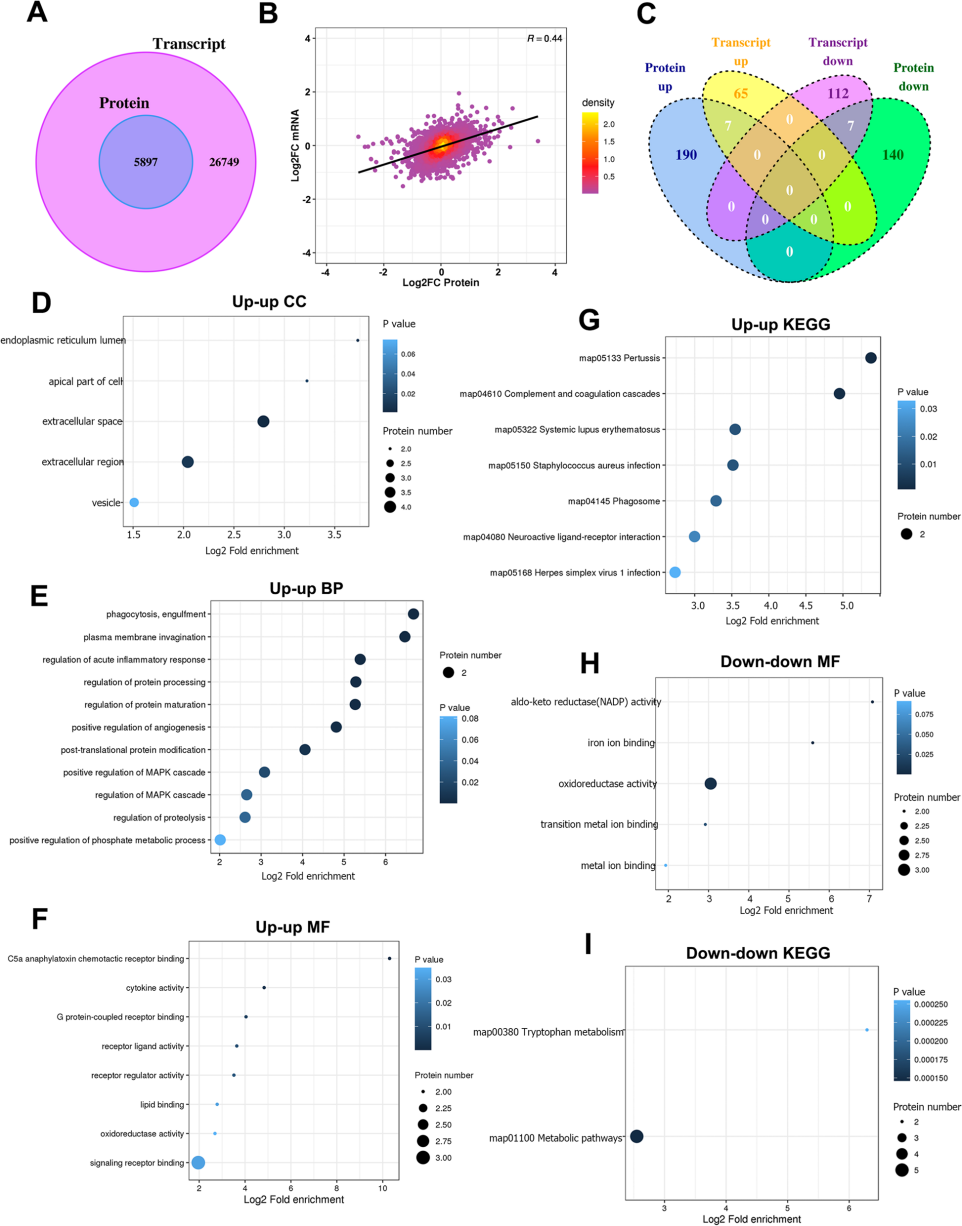
Distinct gene expression profiles identification by integrated proteomic and transcriptomic analysis. **A** Wayne diagram for quantitative comparison of transcriptome and proteome. **B** Scatter plot of transcript and its corresponding protein expression. **C** Wayne diagram analysis of differentially expressed proteins and transcripts. **D** Cellular component of proteins in up-up group. **E** Biological process of proteins in up-up group. **F** Molecular function of proteins in up-up group. **G** KEGG analysis of proteins in up-up group. **H** Molecular function of proteins in down-down group. **I** KEGG analysis of proteins in down-down group

With matched RNA-Seq and proteomics data described above, we set out to assess the integrated proteotranscriptomic analysis at several levels. First, we converted the protein ID into the corresponding transcripts ID, and then analyzed the data of the two omics according to the transcript ID. Results showed that 5 897genes were quantified at both transcriptome and proteome levels (Fig. 6A). RNA abundance and Protein abundance are only partially correlated, which is reflected in the *R* = 0.44 (Fig. 6B).

We performed Wayne diagram to compare the proteins and transcripts according to the transcriptional ID (Fig. 6C), results showed that 7 genes were both upregulated at protein and transcripts level (up-up group), and 7 genes were both down-regulated at protein and transcripts level (down-down group). Particularly, the kidney injure-related factors Havcr1 and Spp1 were both significantly up-regulated at protein and transcript level. Notably, there were 140 genes down-regulated at protein level without significant change at transcript level (down-unchange group), and 190 genes upregulated at protein level with no significant change at transcript level (up-unchange group). A number of solute carriers and transporters including Slc22a22, Slco1a1, Slc21a4, Slc16a4, Slc1a1, Slc22a1, Slc34a1, Slc3a1, Slc22a6 and Slc34a3 were significantly decreased at protein level with no significant change at transcript level.

Further, we analyzed the functional enrichment of the genes consistently changed at both protein and transcript level. Result showed that the genes in the up-up group were most enriched in the extracellular region/space (Fig. 6D), phagocytosis and membrane invagination (Fig. 6E), and C5a anaphylatoxin chemotactic receptor binding (Fig. 6F). The most enriched KEGG signaling pathway was complement and coagulation cascades (Fig. 6G). Nevertheless, genes in the down-down group were most enriched in oxidoreductase activity (Fig. 6H) and involved in the metabolic pathways (Fig. 6I).

In addition, we also found that a number of genes related to protein poly-ADP-ribosylation (Parp10 and Parp14), glutamine catabolic process and glutamate biosynthetic process (Gls) and phagocytosis (Sirpa, Mfge8, Itgb2, Arhgap12, Elmo1 and Sh3bp1) were increased at protein level but unchanged at transcript level. Notably, several genes related to regulation of acute inflammatory response (C1qc, C1qb, Cqb2, Kng1, C8b, C8a, C6, Clu, C9) and immunoglobulin mediated immune response (RT1-Bb, Crp and Lnpp5d) were also up-regulated at protein level but no significant change at transcript level (Supplementary Fig. 3). Our data indicated that post-transcriptional regulation may also play critical roles in the metabolic process and immune response of crystal formation and kidney injury.

### Verification of the DEGs by IHC analysis

**Fig. 7:**
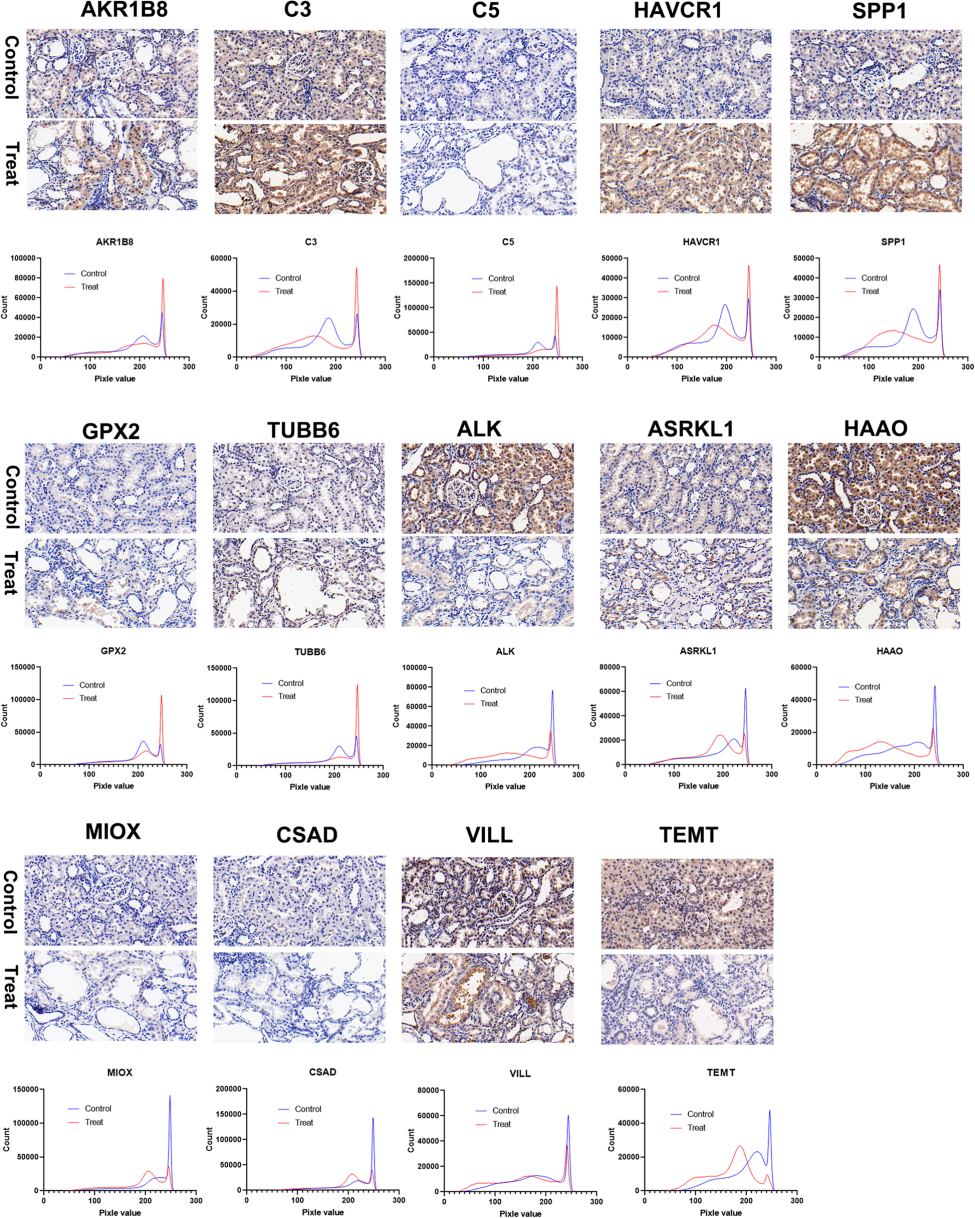
Verification of the DEPs by IHC analysis. Original magnification, × 40. The histogram profile corresponds to the pixel intensity value vs. corresponding number counts of a pixel intensity

For validation, the common expressed genes at mRNA and protein levels were validated by immuno-histochemistry analysis. Spp1, Akr1b8 and Havcr1 were the top three most increased genes in the up-up group. We found that Spp1 was strongly stained in the proximal tubular cells and much higher in the tissue of treat group than that in normal control (Fig. 7). Havcr1 was significantly enhanced expression in the kidney of calculi rats (Fig. 7). Notably, the kidney tubule cell-released circulating Spp1 was correlated with kidney injury in patients [30], and Havcr1 was markedly upregulated to promote phagocytosis and inhibit innate immunity and inflammation via p85-PI3K-NFκB signaling in the proximal tubule cells after kidney injury [33]. Our data indicated that the calculi rats were suffering significant kidney injury during the crystal formation with markedly increased expression of Spp1 and Havcr1. We also found that the complements including C3 and C5 were common up-regulated and higher stained in the kidney of calculi rats (Fig. 7), which promoted phagocytosis, trigger inflammation and immune clearance, played central role in the activation of complement system [34]. Gpx2 belongs to the glutathione peroxidase family, plays a major role in protecting mammals against oxidative damage [35]. Thus, the up-regulation of Gpx2 might result from the oxidative damage in the kidney of calculi rats.

Moreover, we also found that 7 genes were commonly down-regulated at transcript level and protein level and verified by immune-histochemistry analysis (Fig. 7). Of which Haao, Miox, Inmt, Haao, Csad and Alpl were involved in metabolic pathways, and the reduced expression of Akr1c12l1 was associated with oxidoreductase activity. These data confirmed and revealed that the metabolic network activity was significantly reduced in the kidney of calculi rats. Notably, a set of proteins were decreased at protein level with no significant change at transcript level, which potentially regulated by post-transcriptional modification. Our verification study showed that all the immune-histochemistry results were consistent with the findings from transcriptomic analysis.

## Discussions

Calcium oxalate urolith is accounted over 70 percent of all kinds of kidney stone [36], ranked the most common type of urolithiasis in patients worldwide [37, 38]. However, little is known about the mechanism of oxalate calculi crystals formation and calculi-related kidney injury. In present study, we demonstrated that by combining comprehensive RNA-seq data and high-resolution liquid chromatography-mass spectrometry analysis, achieved a better understanding of the profile of protein-coding genes in the kidney of calculi rats than previous efforts based on transcriptome or proteome guided denovo strategies.

We identified 14 protein-coding genes with consistent expression patterns at both protein and transcript levels in the kidney of calculi rat model. In the up-up group, the GO terms of ER lumen and phagocytosis were significantly enriched, which indicated enhanced ER stress and immune response in the kidney of calculi rats. The ER stress may lead to apoptosis, renal injury, and calcium oxalate crystal deposition in the renal of calculi rats via sigma-1 receptor related mitochondria dysfunction and reactive oxygen species (ROS) generation [39–42]. M2-macrophages associated phagocytosis could suppress kidney stone development through serval mechanisms, including NLRP3, miR-93-TLR4/IRF1, and miR-185-5p/CSF1 pathways [43, 44]. Therefore, C3, C5 and HAVCR1 related promotion of M2-like macrophage polarization and inhibition of inflammation could prevent intrarenal CaOx deposits, nucleation and kidney stone recurrence.

We found that the protein SPP1, C3 and HAVCR1 might also play critical roles in the ER stress and phagocytosis in the CaOx crystal formation process and its related kidney injury. In the down-down group, the protein-coding genes mainly enriched in NADP activity, oxidoreductase activity and metabolic pathways, which related to mitochondrial dysfunction and ROS overproduction, and led to cellular injury [45, 46]. Thus, MIOX, Akr1c12l1 and HAAO may act as negative modulators in the mitochondrial dysfunction related calcium oxalate crystal deposition. In addition, several components of vacuolar ATPases responsible for the translocation of H^+^ ions across membranes were also observed decreased expression at protein level in the kidney of calculi rats, suggested that decreased vacuolar ATPase activity may alter the cytoplasmic pH of the leading-edge environment of tubule cells.

We also found that a number of solute carriers and transporters (including Slc34a1 and Slc34a3) were decreased at protein level with no significant change at transcript level. For example, the Slc34a1 (NaPi-IIa) and Slc34a3 (NaPi-IIc) are responsible for filtration of phosphate from primary urine [47], play a crucial role of in calcium metabolism as well as phosphate balance in humans. The Slc22 transporter family are widely studied drag transporters, which regulate key metabolic pathways and optimize levels of numerous metabolites and signaling molecules, as well as uremic toxins associated with many chronic kidney diseases [48–50]. Thus, our results may reflect the functional changes in the tubule cells in the kidney of calculi rats.

Beyond the consistent upregulated C3 and C5, many complements related to regulation of acute inflammatory response were significantly increased at protein level with no change at transcript level in the kidney of calculi rats. Complement and coagulation cascades activation leads to chemotaxis and immune-complex clearance [51], is one of the most significantly enriched signaling pathways in the calcium oxalate crystal-induced ROS in kidney [52]. The deposition of locally produced and activated complement fragments can also drive severe inflammatory response in the kidney and result in complement-mediated inflammatory injury [51]. Notably, the forementioned solute carriers and transporters, as well as complements exhibited inconsistent expression pattern of transcripts and corresponding protein. Which indicated that the ER stress and its related post-transcriptional modification (PTM) might play a role in these protein coding genes transcriptional modulation. To date, over 100 types of mRNA related PTMs have been identified. Modifications at the 5’-cap and the 3’-end poly (A) tail of mRNAs play key roles in regulation, transcript stability, pre-mRNA splicing, polyadenylation, mRNA export, translation initiation and nuclear export, which are gaining increasing attention for their roles in cellular metabolism [53]. For example, the SLC34A1 protein expression level is regulated by specific chromatin architecture and SNPs elements [54], as well as natural antisense transcripts [55]. However, the realm of post-transcriptional gene regulation in kidney stone formation is still far from clear.

Although prevention the occurrence of new calcium stones and removing kidney stones with flexible ureteroscopy are possible today, there is no doubt that recurrence preventions are much more important and need to be further developed [56]. A better understanding of the mechanisms involved in stone formation are absolute prerequisites for kidney stone recurrence prevention. In present study, a series of proteins with distinct expression profiles related to metabolism and immune response has been identified to play critical roles in the kidney stone initiation. For example, C3 and C5 are main factors in complement and coagulation cascades related to the calcium oxalate crystal-induced ROS in kidney. C3 and C5 inhibitors might contribute to attenuate the complement-driven inflammation in kidney stone formation and recurrence. Additionally, beside the mechanism investigation facilitated pharmacological therapy development, individualized recurrence prevention procedures are important aspects for kidney stone prevention.

## Conclusions

**Fig. 8:**
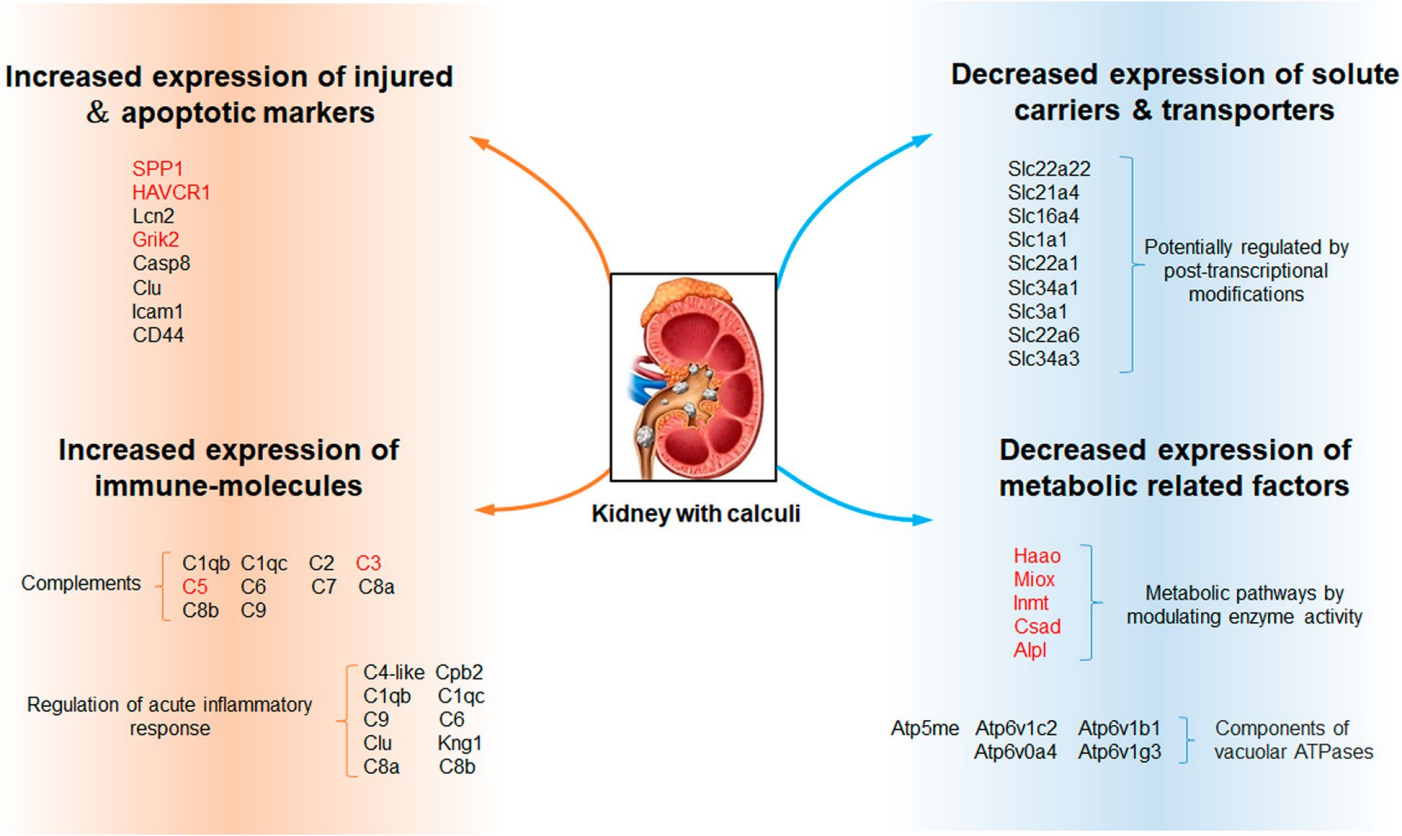
An overview of possible biological changes that might contribute to crystal formation in the kidney of calculi rats

In general, we characterized the calculi oxalate crystals-related gene expression profiles in the kidney by integrated proteomics and transcriptomics analysis. Our results showed that the calculi rat kidney was increased expression of injured & apoptotic markers and immune-molecules. On the other hand, the calculi rat kidney was decreased expression of solute carriers & transporters and many metabolic factors (Fig. 8). These effects jointly contribute to the formation of kidney stones and calculi-related kidney injury. The present proteotranscriptomic study has provided a data resource and new insights for better understanding of the pathogenesis of nephrolithiasis, will hopefully facilitate the future development of new strategies for the recurrence prevention and treatment in patients with kidney stone disease. The main limitation of this study could have been present due to the animal-based oxalate calculi model, which could not perfectly mimic the status of crystals in the kidney of patients. In the future, kidney organoids derived from human pluripotent stem cells might have great utility for kidney stone modeling.

## Supplementary Information

**Additional file 1: Supplementary Table 1.** The FPKM of genes analyzed in the kidney of rats.

**Additional file 2: Supplementary Table 2.** The MS information of proteins identified in kidney of rats.

**Additional file 3: Supplementary Table 3.** Details of DEGs in the kidney of calculi rats compared to normal control.

**Additional file 4: Supplementary Table 4.** Details of DEPs in the kidney of calculi rats compared to normal control.

**Additional file 5: Supplementary Fig. 1.** Cluster analysis of differentially expressed genes and protiens. (A) Heatmap of DEGs. (B)Heatmap of DEPs.

**Additional file 6: Supplementary Fig. 2.** Functional enrichment analysis of differentially expressed protiens. (A) Up GO. (B) Down GO. (C) UP KOG. (D) Down KOG.

**Additional file 7: Supplementary Fig. 3.** Heatmap of functional enrichment analysis of proteins under different regulatory relationships between transcriptome and proteome.

## Data Availability

The mass spectrometry proteomics data have been deposited to the ProteomeXchange Consortium (http://www.ebi.ac.uk/pride) via the PRIDE partner repository with the dataset identifier PXD039169.

## Abbreviations

NHANES: National Health and Nutrition Examination Survey
MGP: Matrix Gla protein
THP: Tamm-Horsfall proteins
COM: Calcium oxalate monohydrate
ER: Endoplasmic reticulum
SD: Sprague–Dawley
HE: Hematoxylin–Eosin
GO: Gene Ontology
DEGs: Differentially expressed genes
DEPs: Differentially expressed proteins
KEGG: Kyoto Encyclopedia of Genes and Genomes
PCA: Principal component analysis
GPRIN: G-protein-regulated inducer of neurite
ALI: Acute lung injury
AKI: Acute kidney injury
ROS: Reactive oxygen species
