# Supplementary figures and images for: Proteomics and transcriptomics profiling reveals distinct aspects of kidney stone related genes in calculi rats
Source: BMC Genomics. 2023 Mar 17;24:127. doi: 10.1186/s12864-023-09222-7 (PMC10024419; doi:10.1186/s12864-023-09222-7)

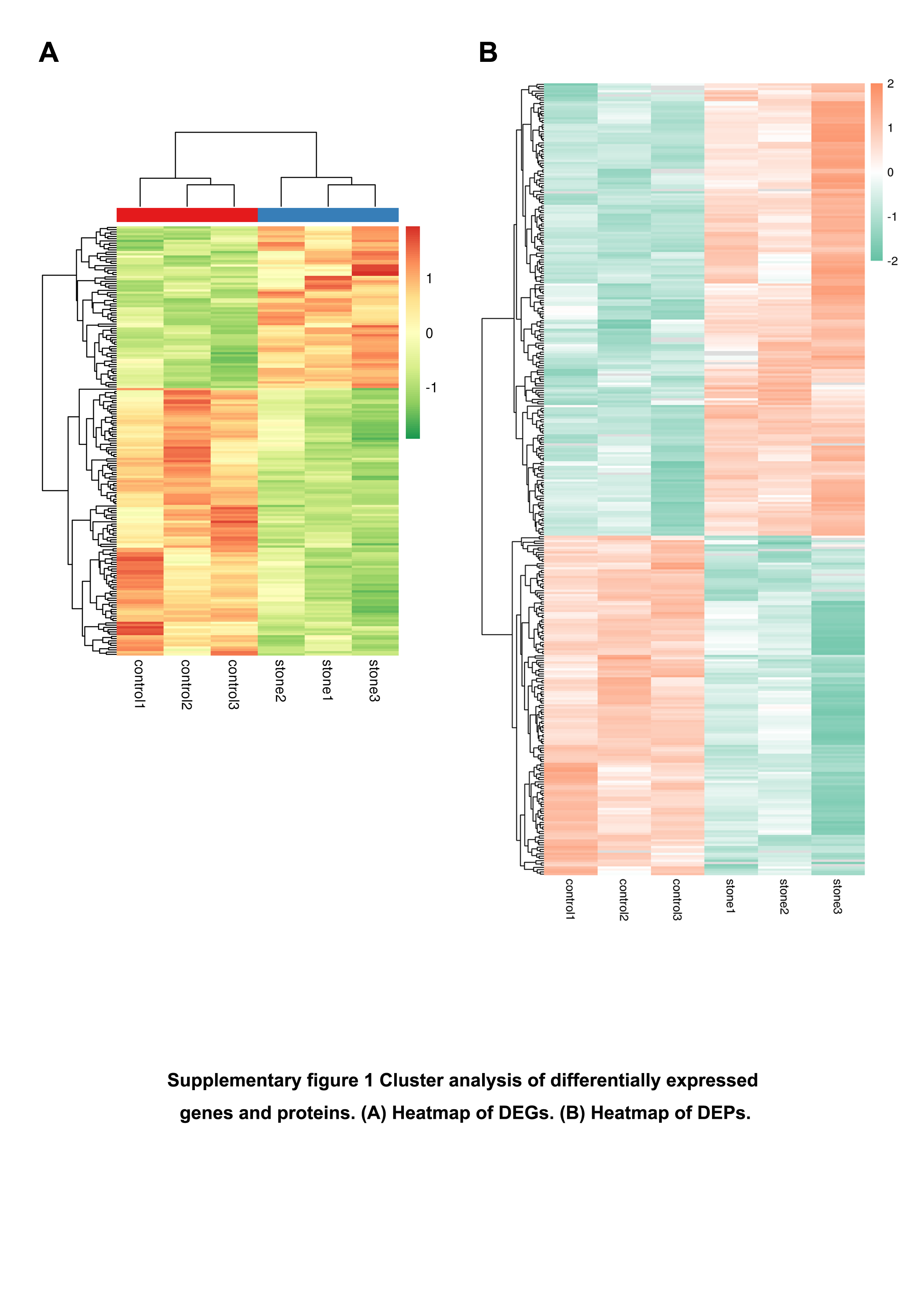

Supplement: Supplementary file 5 — Additional file 5: Supplementary Fig. 1. Cluster analysis of differentially expressed genes and protiens. (A) Heatmap of DEGs. (B)Heatmap of DEPs. [file 12864_2023_9222_MOESM5_ESM.tif]

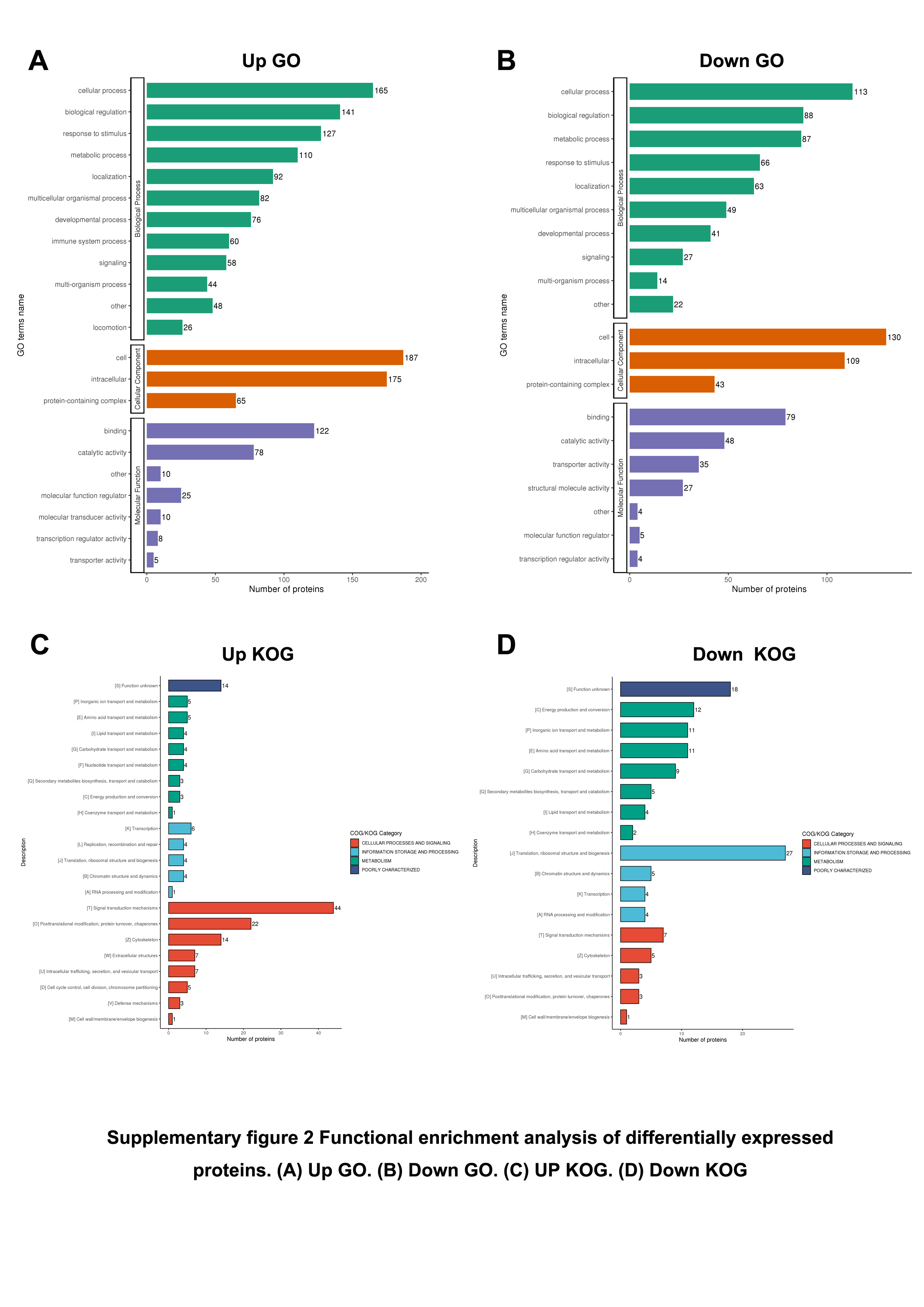

Supplement: Supplementary file 6 — Additional file 6: Supplementary Fig. 2. Functional enrichment analysis of differentially expressed protiens. (A) Up GO. (B) Down GO. (C) UP KOG. (D) Down KOG. [file 12864_2023_9222_MOESM6_ESM.tif]

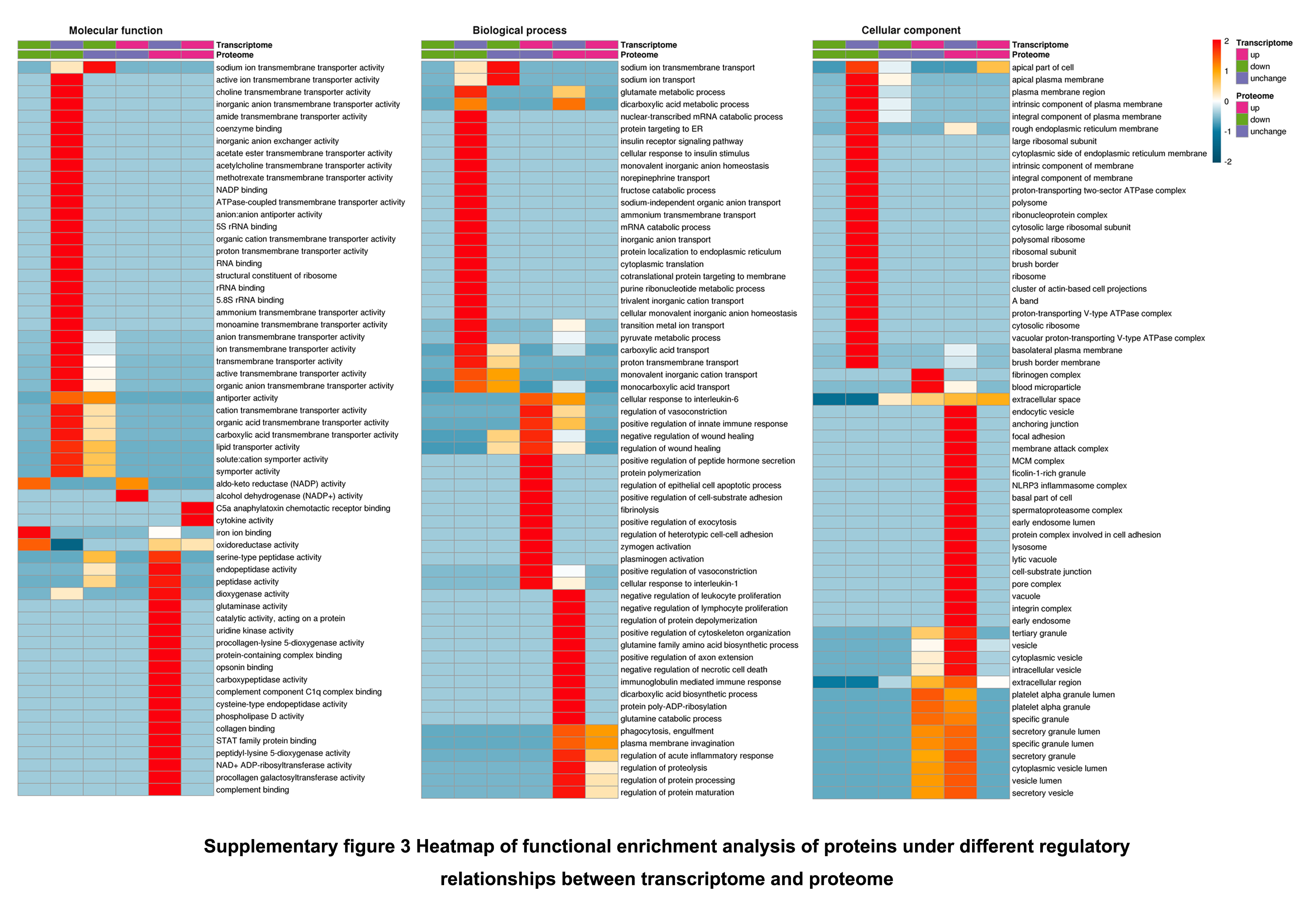

Supplement: Supplementary file 7 — Additional file 7: Supplementary Fig. 3. Heatmap of functional enrichment analysis of proteins under different regulatory relationships between transcriptome and proteome. [file 12864_2023_9222_MOESM7_ESM.tif]
